# Adrenal Insufficiency under Standard Dosage of Glucocorticoid Replacement after Unilateral Adrenalectomy for Cushing's Syndrome

**DOI:** 10.1155/2016/2347528

**Published:** 2016-06-07

**Authors:** Kentaro Fujii, Kazutoshi Miyashita, Isao Kurihara, Ken Hiratsuka, Seiji Sato, Kenichi Yokota, Sakiko Kobayashi, Hirotaka Shibata, Hiroshi Itoh

**Affiliations:** ^1^Department of Internal Medicine, School of Medicine, Keio University, 35 Shinanomachi, Shinjuku-ku, Tokyo 160-8582, Japan; ^2^Department of Endocrinology, Metabolism, Rheumatology and Nephrology, Faculty of Medicine, Oita University, 700 Dannoharu, Oita 870-1192, Japan

## Abstract

Glucocorticoid replacement is needed for patients after adrenal surgery for Cushing's syndrome; however, the adequate dosage is not easily determined. The patient was a 62-year-old woman who has had hypertension for 5 years and presented with heart failure due to hypertrophic cardiomyopathy. She consulted with us because of general fatigue, facial edema, and muscle weakness and was diagnosed with Cushing's syndrome. A laparoscopic left adrenalectomy was performed, standard dosage of postoperative replacement was administered, and she was discharged with 30 mg/day of hydrocortisone (cortisol). However, she suffered from loss of appetite and was transferred to an emergency unit with the symptoms of adrenal insufficiency on postoperative day 15. After initial hydrocortisone replacement with 200 mg/day, the dosage was gradually decreased during hospitalization; however, reduction of hydrocortisone dosage lower than 60 mg/day was difficult because of nausea and fatigue. Her circadian cortisol profile after hydrocortisone administration showed delayed and lowered peaks, which suggested that hydrocortisone absorption in the intestine was impaired. Therefore, complicated heart failure may have led to the adrenal insufficiency in the patient. In such cases, we should consider postoperative administration of more than the standard dosage of hydrocortisone to avoid adrenal insufficiency after surgery for Cushing's syndrome.

## 1. Introduction

The patients who have undergone a unilateral adrenalectomy for Cushing's syndrome become steroid-dependent. Therefore, sufficient replacement of glucocorticoid is needed during the postoperative period [1, 2]. Although a standard protocol for postoperative replacement has not been developed yet, the dosage was empirically suggested by some previous reports. For example, a 200 mg dose of hydrocortisone was administered within the first 24 hours after surgery; thereafter, 100 mg every 8 hours for a day, 100 mg every 12 hours for a day, and 50 mg every 12 hours for a day were applied during the following three days, respectively. On the fourth day, an oral dose of 25 mg hydrocortisone was subsequently used instead of intravenous agents, followed by a reduction of 5 mg every 3 days until a maintenance dose (15–20 mg/day) [3].

Previous reports indicate that patients with Cushing's syndrome who undergo a unilateral adrenalectomy can usually be tapered off of all steroids within 6 months to 2 years; however, the adequate dosage and duration are not easily determined [4–6]. Moreover, some patients suffer from glucocorticoid withdrawal symptoms and need an increase in the glucocorticoid dose. Recently, the dosage of postoperative glucocorticoid replacement has a trend to be reduced to help the recovery of adrenal function after adrenalectomy [1]. However, it should not be always the case. Here we show an instructive case of a patient who suffered from symptoms of adrenal insufficiency after a unilateral adrenalectomy for Cushing's syndrome, which occurred despite treatment with more than 30 mg/day of hydrocortisone.

## 2. Case Presentation

A 62-year-old Japanese woman consulted with us because of progressively worsening general fatigue, facial edema, and muscle weakness. She has suffered from hypertension for 5 years and recognized the easy bruising, edematous face, and loss of muscle strength. Past medical history showed heart failure due to hypertrophic cardiomyopathy and hepatitis B virus (HBV) infection. Her medications included furosemide, spironolactone, bisoprolol, verapamil, cibenzoline, lovastatin, and entecavir. No family history of hypertension was recorded.

Physical examination showed normal vital signs with a blood pressure of 127/86 mmHg and a pulse rate of 74 bpm. Her BMI was 23.2 kg/m^2^. Heart sounds were clear and regular without murmurs. Breath sounds were also clear. Pitting edema was observed in the lower extremities. Moon-shaped face and buffalo hump were also present. Laboratory findings revealed mild renal dysfunction [creatinine 1.03 mg/dL (0.60–1.20 mg/dL), blood urea nitrogen 34.4 mg/dL (7–20 mg/dL)] and fluid retention [brain natriuretic peptide (BNP) 547.6 pg/mL (<18.4 pg/mL)]. Serum electrolytes were within normal limits [potassium 4.7 mmol/L (3.6–5.0 mmol/L), sodium 144.9 mmol/L (136–146 mmol/L), and chloride 105 mmol/L (97–107 mmol/L)]. Electrocardiogram (ECG) and ultrasound cardiography (UCG) revealed that hypertrophic cardiomyopathy was present in the left ventricle but it did not obstruct the ventricular outflow tract. The inferior vena cava was not distended.

**Figure 1:**
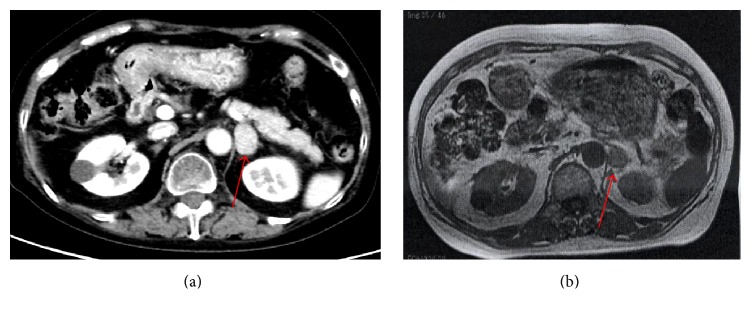
A 62-year-old woman with Cushing's syndrome: (a) computed tomography and (b) magnetic resonance imaging showed an adrenal tumor (29 × 22 mm, red arrow).

Endocrinological evaluations revealed elevated urinary free cortisol (114 *μ*g/day), without suppression of serum cortisol at midnight (18.1 *μ*g/day) and with suppression of ACTH (<1.0 pg/mL) in the morning. After an overnight 8 mg dexamethasone challenge, the serum cortisol level was maintained at 34.4 *μ*g/dL and ACTH was <1.0 pg/mL in the next morning. Abdominal computed tomography (CT) and magnetic resonance imaging (MRI) detected a 30 mm left adrenal mass (Figure 1) and 131I-adosterol scintigraphy revealed unilateral uptake in the left adrenal mass. Under a diagnosis of Cushing's syndrome caused by an adrenal adenoma, a laparoscopic left adrenalectomy was performed and 200 mg/day of hydrocortisone was started postoperatively. The dose was reduced gradually and she was discharged on postoperative day 14 with 30 mg/day of oral hydrocortisone.

**Figure 2:**
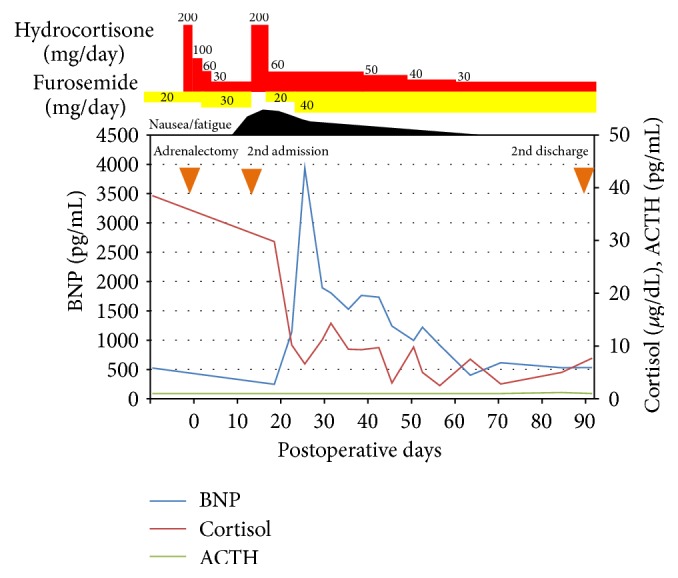
Clinical course of the patient after the operation. The brain natriuretic peptide (BNP) level (blue line) is shown on the left side. The levels of cortisol (red line) and ACTH (green line) are shown on the right side. The dosages of hydrocortisone and furosemide are demonstrated on the upper side.

However, she felt severe nausea and fatigue just after discharge and was transferred to an emergency unit on postoperative day 15 (Figure 2). Her blood pressure had declined to 95/64 mmHg, hyponatremia [sodium 138.6 mmol/L (136–146 mmol/L)] was observed, and ACTH was undetectable. From these facts, she was diagnosed with adrenal insufficiency. The symptoms immediately disappeared by an intravenous infusion of 200 mg hydrocortisone. After the initial treatment, she felt dyspnea and her BNP level was remarkably increased to 3934.5 pg/mL (<18.4 pg/mL). Under the diagnosis of acute exacerbation of heart failure, furosemide was increased to treat the fluid retention and the BNP level gradually decreased. The signs and symptoms of acute coronary syndrome were not observed.

**Figure 3:**
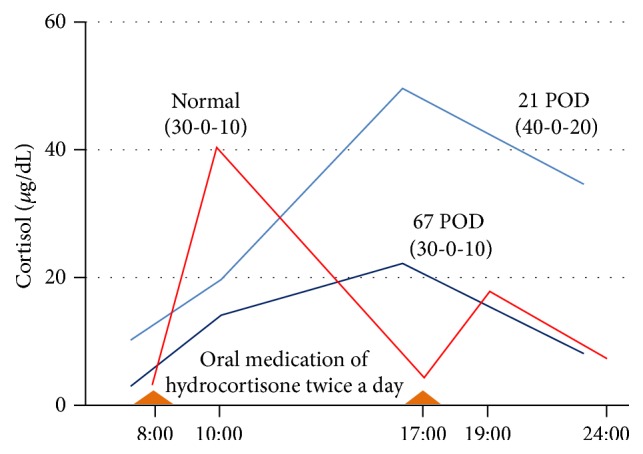
Delayed and lowered peaks of serum cortisol level after oral medications of hydrocortisone. The serum cortisol levels of the patient on 21 and 67 postoperative days (POD) are shown (blue lines). The peak of cortisol of the patient after oral medication of hydrocortisone showed substantial delay when compared to normal control (red line). The levels of normal control are cited from previous reports [15].

The dose of hydrocortisone was slowly decreased under hospitalization; however, reduction of hydrocortisone lower than 60 mg/day was difficult because of nausea and fatigue. We suspected that the heart failure caused malabsorption of the hydrocortisone, because her circadian profile of cortisol showed delayed and lowered peaks after hydrocortisone administration (Figure 3). The daily dose of hydrocortisone was carefully decreased from 60 mg/day, in accordance with her symptoms. However, she presented fatigue and loss of appetite which correlated with the dose of hydrocortisone and it was difficult to reduce the dosage until the standard maintenance dose (15–20 mg/day). Finally, she was discharged on postoperative day 92 at a dose of 30 mg/day, because the nausea and fatigue had disappeared and she resumed daily life activities. Three years after the operation, her hypothalamus-pituitary-adrenal (HPA) axis has completely recovered.

## 3. Discussion

Cushing's syndrome was first described by Cushing in 1932 [7] and it is currently classified as ACTH dependent or independent. The syndrome is defined as an endocrine disorder with a constitutively elevated level of glucocorticoids. The patients present with central obesity, diabetes mellitus, osteoporosis, and other metabolic symptoms. In general, patients who have undergone a unilateral adrenalectomy for Cushing's syndrome become steroid deficient; therefore, they absolutely need postoperative replacement therapy [2]. A clinical practice guideline stated that glucocorticoid replacement after surgery is required until the HPA axis recovers and the mean period of replacement is eighteen months after a unilateral adrenalectomy [1].

Although the standardized protocol for glucocorticoid replacement during the perioperative period was not mentioned in the guideline, some previous reports showed recommended protocols. For example, Orth and Kovaks stated that 200 mg of hydrocortisone should be given within the first 24 hours after surgery, and then 100 mg, 75 mg, and 50 mg/day on the following days with a gradual reduction of hydrocortisone to a maintenance dose (15–25 mg/day) [8]. Since steroid withdrawal syndrome (SWS) may happen when the dosage of glucocorticoid is decreased too quickly, even after the HPA axis has begun to recover, the dosage should be tapered down slowly or a temporary increase may be needed [1]. The mechanism of SWS is still unknown; however, it is assumed that long-term exposure to glucocorticoids causes the patients to develop a dependence on glucocorticoids [9].

In our case, a standard steroid replacement with 30 mg/day of hydrocortisone was not enough to avoid adrenal insufficiency after a unilateral adrenalectomy for Cushing's syndrome. The patient had presented with the typical clinical features of Cushing's syndrome for five years and was exposed to excessive cortisol for a long time. Chronic exposure to excessive glucocorticoids is known to impair biological effects due to downregulation of the receptor [10]. Therefore, decreased glucocorticoid receptors may have caused her to need more hydrocortisone than the standard dose. However, it does not explain the delayed cortisol level peaks after hydrocortisone administration (Figure 3).

We considered that “worsening of heart failure due to adrenal insufficiency” after the second admission would be an important factor for the increase in the required amount of glucocorticoids. Hemodynamic impairment, caused by volume depletion and low cardiac output, is a common problem in adrenal insufficiency [11]. Several reports have shown structural myocardial changes during adrenal insufficiency, such as stress cardiomyopathy, as well as a rapid recovery under steroid therapy [12, 13]. An excessive amount of crystalloid solutions, which was used for fluid resuscitation against volume depletion and lower blood pressure in adrenal insufficiency, might be an exacerbating factor for the heart failure. Since the patient had hypertrophic cardiomyopathy and a higher baseline BNP level before surgery, the heart failure might be much easier to exacerbate for the above reasons. Furthermore, intestinal edema and reduced absorption of intestinal contents are well known problems in patients with heart failure [14]. In this case, the delayed and lowered peaks of cortisol level after hydrocortisone administration suggested malabsorption in the intestine. In these contexts, we judged that adrenal insufficiency of the patient on second admission triggered the exacerbation of heart failure which led to malabsorption of hydrocortisone. That is, adrenal insufficiency after surgery made heart failure and malabsorption more serious and formed a vicious cycle. In such cases with heart failure, a hydrocortisone replacement of more than a 30 mg/day dose should be considered after a unilateral adrenalectomy for Cushing's syndrome.

## 4. Conclusion

We presented a case of a patient with adrenal insufficiency after a unilateral adrenalectomy for Cushing's syndrome who was resistant to a reduction in glucocorticoid replacement. The glucocorticoid receptors would have been downregulated due to the long-term exposure of excessive cortisol before surgery. It was suggested that complicated heart failure reduced the absorption of hydrocortisone in the intestine. We judged that adrenal insufficiency after surgery exacerbated the heart failure and malabsorption of hydrocortisone which formed a vicious cycle. In cases with heart failure, enough hydrocortisone replacement with a dose of more than 30 mg/day should be considered to avoid adrenal insufficiency after a unilateral adrenalectomy for Cushing's syndrome.
